# Supplementary figures and images for: Genome-wide insights into adaptive hybridisation across the Schistosoma haematobium group in West and Central Africa
Source: PLoS Negl Trop Dis. 2022 Jan 31;16(1):e0010088. doi: 10.1371/journal.pntd.0010088 (PMC8803156; doi:10.1371/journal.pntd.0010088)

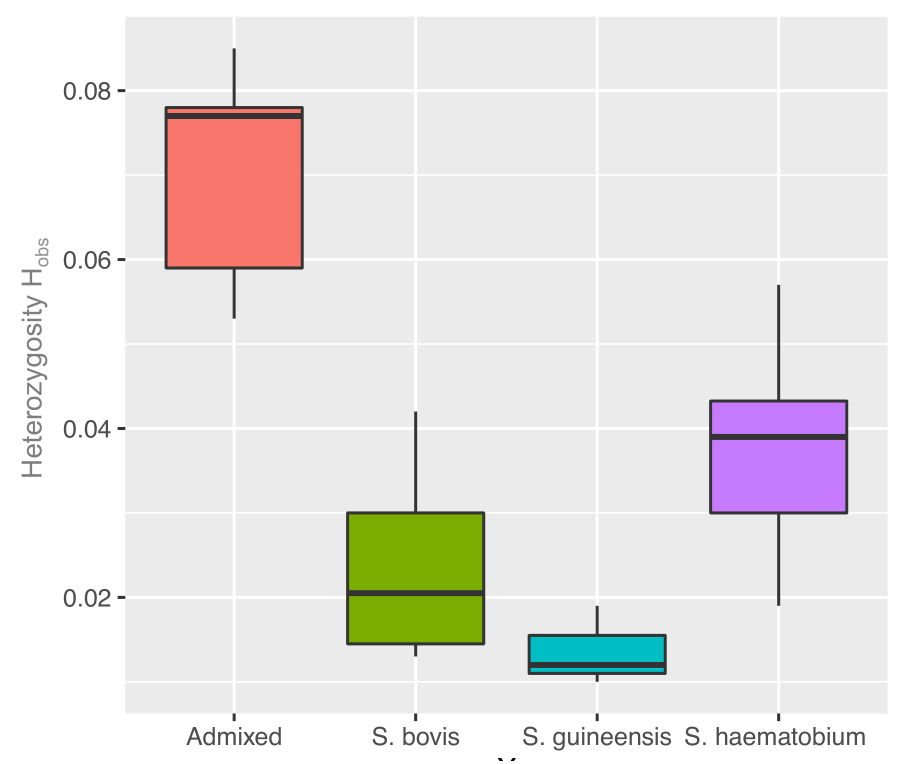

Supplement: S2 Fig — (TIFF) [file pntd.0010088.s002.tiff]

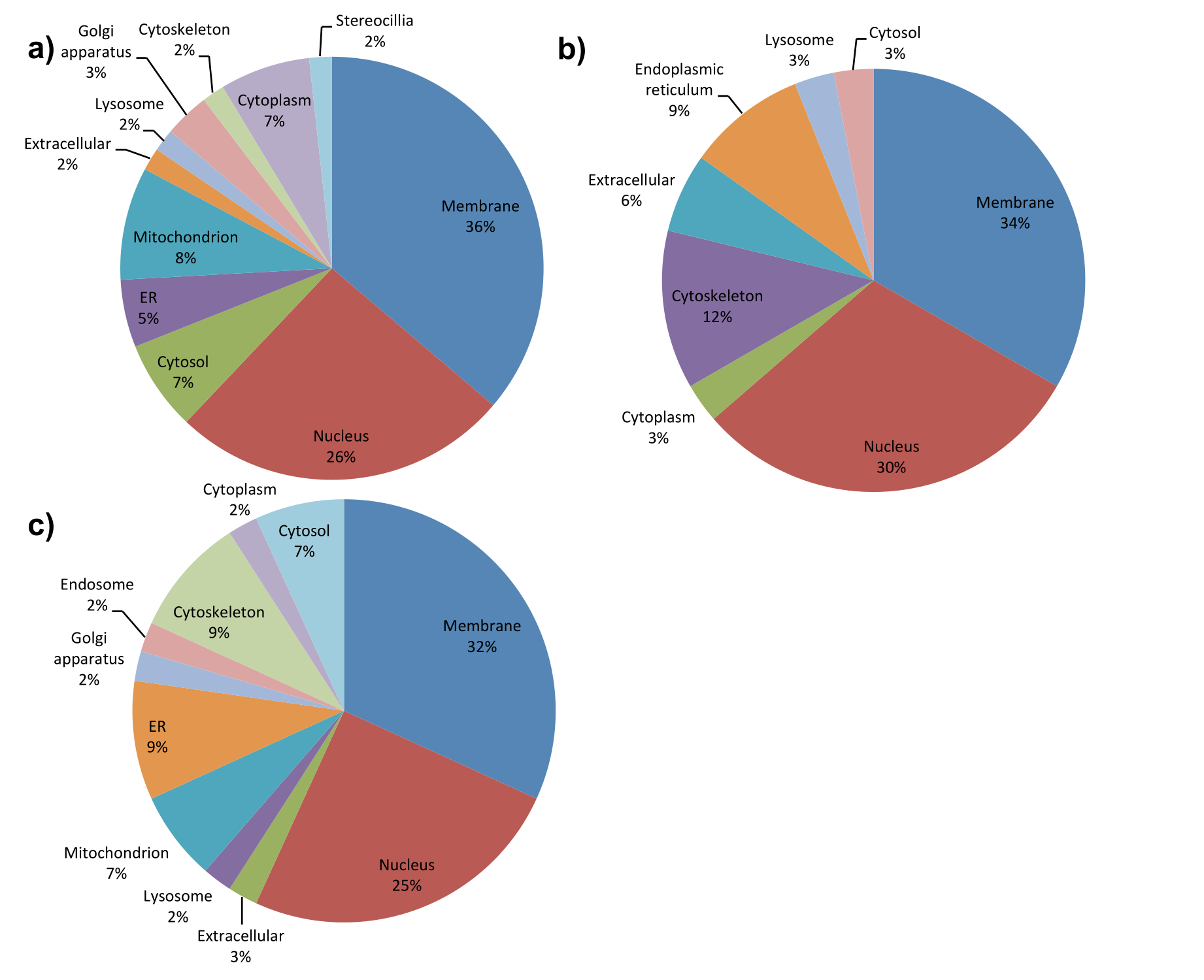

Supplement: S4 Fig — The translation and functionality is established via the WormBase ParaSITE database. Each pie chart describes cellular locations of outliers described in species comparisons across; a) S. haematobium x S. bovis, b) S. haematobium x S. guineensis, c) S. guineensis x S. bovis. (TIFF) [file pntd.0010088.s004.tiff]
