# Supplementary material for: Genome-wide insights into adaptive hybridisation across the Schistosoma haematobium group in West and Central Africa
Source: PLoS Negl Trop Dis. 2022 Jan 31;16(1):e0010088. doi: 10.1371/journal.pntd.0010088 (PMC8803156; doi:10.1371/journal.pntd.0010088)
Supplement: Attachment — Submitted filename: Reviewers Responses.docx [file pntd.0010088.s010.docx]

**Reviewers Responses**

**Summary and General comments**

Reviewer #1

The manuscript reads well throughout though some sections starting with the introduction would benefit from some English editing, as they are not easy to understand, which impacts the subsequent flow of the manuscript.

English edits and ammendments have been made throughout the document

L85-86, I believe S. haematobium is reported to cause urogenital schistosomiasis disregarding of whether it occurs in an endemic or non-endemic area.

Ammended sentence L83-86

Consider explaining to readers new in this field what hybridisation or introgression actually means also from a genomic point of view.

Ammended; L144 – L155

“Introgression refers to the exchange of genes between divergent lineages that results from backcrossing of hybrids with one or both parents. Also described as hybridization, this should result in a relatively even mixture of both gene and allele frequencies deriving from both parental species [20]. The genomes of hybrids are characterized by large amounts of genetic variation compared to single species parental counterparts, however hybrid genomes regularly contain novel genetic combinations that may have functional consequences [21]. Patterns of introgressive hybridisation in schistosoma has typically been documented using a limited set of markers [4,5,16,22]. As yet no study has yet investigated the dynamics of locus-specific introgressive hybridisation. This limitation has hampered the detailed understanding of the genomic dynamics of introgression, and the role it plays in hybrid populations of schistosoma parasites”

Also, the potential impact of schistosomal hybridisation on control and prevention measures isn't explained sufficiently to highlight the importance/concerns of such hybrids. Are they not treatable with PZQ?

Ammended; L106 – 143

“Efficacy of praziquantel on hybrid and mixed infections within humans remains strong [13,14], hybridisation events would directly impact current monitoring, evaluation and mapping of the disease within endemic areas [15,16]. Laboratory experiments have demonstrated that F_1_ and F_2_ hybrids between *S. guineensis* and *S. haematobium* exhibited a greater ability to infect snail intermediate hosts along with increased growth rate, reproductive potential and longevity [13–15]. The increased infection potential of hybrids across multiple species of freshwater snail host will increased hybrid populations ability to establish diseases within environments, increasing infection range.”

The paper has already sold hybrids as a source of refugia which will reduce effectiveness of control/elimination programmes. Also introgression has caused the replacement of guineensis with haematobium in some areas, and we need to understand how such processes work, and what, if anything, has been gained by haematobium through the interaction. This is less about drug resistance and more about the epidemiological ramifications which are the backbone control/elimination programmes.

Most importantly as this is the backbone of all your analyses, please explain in more detail your source isolates, i.e. field isolates from individuals and snails from which parts of the countries listed, single species isolates, admixed isolates

The country, location and species description is shown in Fig 1

To which schistosomal stage does the term "isolate" refer to? Why did you look into adult males only? Also, some literature states that larval stages passaged through laboratory intermediate and definitive hosts is prone to artificial selection of specific genotypes

Ammended L227 – 240

“Samples used for sequencing were adult, male, schistosome worms due to DNA yield requirements for RADseq. Field collected isolates refer to infected freshwater intermediate snails, which has then been passaged through laboratory definitive rodent hosts to generate adult worms. Adult worms, stored long-term in liquid nitrogen (approx. -180°C), were thawed and rehydrated for extraction and library preparation workflows. The long term passage of schistosoma worms within a laboratory setting can lead to increased selective advantage of specific genotypes [37]. To mitigate this, samples used within this study were selected from worms derived from first passage; this ensures samples used are true representation of field-derived genotypes. Field isolates were acquired between 1983 and 2006 (Supplementary Table 1) from Nigeria, Cameroon, Senegal, Mali and the islands of São Tomé (Fig 1). Adult worms were stored long-term in liquid nitrogen (approx. -180°C), thawed and rehydrated for extraction and library preparation.”

Please put more emphasis on describing your source isolates and if possible simplify the description of your analytical part.

I have added additional information regarding the samples used, source of said samples and reasoning behind the sample selection. I don’t believe it is possible to reduce the description of the analytical methods used in this study. This study is the first time that RADseq has been used on schistosoma parasites. This is also the first time that the specific analytical pipeline has been used to determine locus-specific selection, which occurs in schistosoma hybrids. This requires a full and detailed explanation of specific calculations used and why they are the correct ones to use for the study.

**Results**

Reviewer #2:

Fig 5 looks fancy, however, it's difficult to interpret for a general audience. Therefore, I would suggest it might be better to move figure 5 to supplementary data and move the S 4 Table (showing list of identified antigens/proteins), which is more obvious and informative to the main table of the manuscript.

Fig 5 is a integral figure which displays the results from bayescan pipeline, this would need to be included to show the output from bayescan genome-wide selection analysis, I have expanded the figure legend to better help the general audience understand the plots. Supplementary table 4 a descriptive table that includes many columns, which support Figure 5, I don’t believe there, is a way to display this in the manuscript without deleting columns from the table which are better served as supplementary to the analysis.
